# Controlled One-Step Synthesis of Monodisperse CeO_2_ Octahedra in a Binary Solvent System with Waste Liquid Recycling

**DOI:** 10.3390/nano16010053

**Published:** 2025-12-30

**Authors:** Yaohui Xu, Yu Hu, Nengwei Zeng, Haimei Wang, Yuan Zhang, Zongjie Liu, Xinrui Chen, Zhao Ding

**Affiliations:** 1 Laboratory for Functional Materials, School of New Energy Materials and Chemistry, Leshan Normal University, Leshan 614000, China; xyh1986@lsnu.edu.cn (Y.X.); huyu82@lsnu.edu.cn (Y.H.); zllhb1017@163.com (N.Z.); lingping8196@163.com (H.W.); zy112504@163.com (Y.Z.); lzj092800@163.com (Z.L.); cxr041018@163.com (X.C.); 2 Leshan West Silicon Materials Photovoltaic and New Energy Industry Technology Research Institute, Leshan 614000, China; 3 National Engineering Research Center for Magnesium Alloys, College of Materials Science and Engineering, Chongqing University, Chongqing 400044, China

**Keywords:** CeO_2_, octahedra, solvothermal synthesis, morphology control, solvent recycling

## Abstract

To overcome the limitations of template-dependent and anion-assisted methods, this work presents a solvent-controlled strategy for the one-step solvothermal synthesis of octahedral CeO_2_. Using only Ce(NO_3_)_3_·6H_2_O in methanol/water (MeOH/H_2_O) mixtures without the addition of auxiliary templates or surfactants, phase-pure cubic CeO_2_ was obtained. Well-defined octahedra were exclusively formed in a 15 mL MeOH/5 mL H_2_O system at 180 °C for 12 h, whereas other alcohols (including ethanol (EtOH), *n*-propanol (*n*-PrOH), and iso-propanol (*i*-PrOH)) yielded irregular aggregates. Time-dependent evolution revealed continuous crystallinity optimization between 3 and 24 h, beyond which surface dissolution occurred. The solvothermal mother liquor could be recycled four times without compromising phase purity or octahedral morphology, as confirmed by XRD and SEM. This work provides a green and practical route for morphology-controlled oxide synthesis while significantly reducing solvent consumption.

## 1. Introduction

Ceria (CeO_2_) with a cubic fluorite-type structure is the most stable oxide of the rare earth element cerium (Ce), as well as one of the most reactive rare earth metal oxides. CeO_2_ has been extensively researched due to its wide applications as promoters in the three-way catalysts (TWCs) to eliminate volatile organic compounds (VOCs) [1], as oxygen sensors or solid electrolytes in the solid oxide fuel cells (SOFCs) based on their oxygen storage capacity (OSC) [2], as adsorbents for bonding In^3+^, Ga^3+^ and As^5+^ [3] ions or anionic organic dyes [4], and as barriers against ultraviolet radiation [5].

CeO_2_ with a specific size and morphology is preferred due to its remarkable chemical and physical properties. Consequently, increasing efforts have been focused on controlling the morphological characteristics of CeO_2_ micro/nanocrystals, including fine sphere, rod, wire, tube, dendrite, spindle and 3D-flower shapes. Generally, CeO_2_ with specific morphologies can be synthesized using “hard” or “soft” templates. The utilization of “hard” templates primarily involves metal oxide (Al_2_O_3_ [6] and Cu_2_O [7]), mesoporous silica (MCM-48 [8], MCM-41 [9], KIT-6 [10] and SBA-15 [11]), carbon/polymer-based templates (mesoporous carbon [12] and active carbon [13], carbon nanotubes [14] and chitosan film [15]), while the “soft” templates predominantly consist of surfactants (cetyltrimethylammonium bromide, CTAB [16]), biomolecules (amino acids [17], powdered cellulose [18], egg-shell membrane [19]) and polymers (polymethylmethacrylate, PMMA [20]; polyvinyl alcohol/polyvinylpyrrolidone, PVA/PVP [21]; polystyrene spheres [22]). In this way, tedious procedures were required to remove these. In this manner, laborious procedures were necessary to eliminate these “hard” or “soft” templates. This has spurred the development of alternative green synthesis routes, such as water-based systems that enable morphological control without the use of surfactants [23].

Recently, there has been a growing interest in the template- and surfactant-free synthesis of CeO_2_ [24,25,26,27,28,29]. However, it is essential to control the growth of CeO_2_ crystals by adding specific anions that act as capping reagents, such as OH^−^ from NaOH [30] or NH_4_OH [31], NO_3_^−^ from NaNO_3_ [32], S_2_O_8_^2−^ from (NH_4_)_2_S_2_O_8_ [33], or PO_4_^3−^ from Na_3_PO_4_ [34]. Specifically, urea has been commonly used in the hydrothermal synthesis of CeO_2_ [35,36,37]. While effective, such anion-assisted strategies introduce practical and sustainability concerns. The post-synthesis removal of these ions adds operational complexity, increases energy and time costs, and raises the risk of residual anions adversely affecting the catalytic or functional properties of the final CeO_2_ product. Furthermore, the consumption of auxiliary chemicals and the generation of associated wastewater contradict the principles of green chemistry and scalable, sustainable manufacturing.

Hence, we had developed a cost-effective and environmentally friendly one-step solvothermal method for the synthesis of unique octahedral CeO_2_ that eliminates the need for any external templates, surfactants, or controlling anions. The entire reaction system consisted solely of a cerium source (Ce(NO_3_)_3_∙6H_2_O) and a mixed solvent (MeOH/H_2_O). This approach, which uses only the binary solvent without additional shape-directing agents, not only simplifies the process but also inherently avoids the contamination and purification challenges associated with auxiliary agents. Moreover, we integrate a closed-loop solvent recycling strategy, significantly reducing waste generation and enhancing the overall sustainability profile of the synthesis.

## 2. Materials and Methods

### 2.1. Materials

Ce(NO_3_)_3_**∙**6H_2_O was obtained from Aladdin Co. Ltd. (Shanghai, China). Methanol (MeOH), ethanol (EtOH), *n*-propanol (*n*-PrOH) and iso-propanol (*i*-PrOH) were supplied by Chengdu Kelong Chemical Co., Ltd. (Chengdu, China). All reagents were utilized without further purification. Purified water (H_2_O), obtained from an HRO-402 ultrapure water system (Chengdu Zhonghan Water Treatment Equipment Co., Ltd., Chengdu, China), was employed in all experiments conducted throughout the study.

### 2.2. Synthesis of CeO_2_ via One-Step Solvothermal Method

CeO_2_ was synthesized based on a straightforward solvothermal process without any post-treatment calcination. In brief, 4 mmol of Ce(NO_3_)_3_·6H_2_O was dissolved into a 20 mL binary solvent mixture composed of an organic solvent (MeOH, EtOH, *n*-PrOH, or *i*-PrOH) and H_2_O. The mixture was then transferred into a sealed 50 mL Teflon-lined stainless-steel autoclave and heated at 180 °C for a specified duration. After the reaction, the precipitates were collected, washed repeatedly with H_2_O and EtOH, and finally, a series of CeO_2_ powders were obtained by following drying in air at 80 °C for 24 h.

To systematically investigate the effects of solvent composition, a series of experiments was designed as summarized in Table 1. This included: (1) syntheses using pure organic solvents for comparison; (2) syntheses using binary mixtures with a fixed organic solvent/H_2_O volume ratio of 15:5 mL; (3) a study on the MeOH/H_2_O volume ratio with a constant total volume; (4) a study on the total volume with a constant H_2_O amount; and (5) the recycling experiments of the mother liquor.

To assess the sustainability of the process, the solvothermal mother liquor was recycled over multiple cycles. After each synthesis conducted under standard conditions (4 mmol Ce(NO_3_)_3_·6H_2_O in 15 mL MeOH and 5 mL H_2_O, 180 °C for 12 h), the mother liquor was collected and allowed to stand undisturbed for 12 h. The supernatant was then carefully decanted and reused directly as the solvent for the subsequent cycle, without any purification or supplementation. In each new cycle, 4 mmol of fresh Ce(NO_3_)_3_·6H_2_O was dissolved into the recycled supernatant, and the mixture was subjected to the same solvothermal conditions. This recycling procedure was repeated successively for up to eight cycles. The 12 h settling step was implemented to minimize the carryover of residual nanocrystalline nuclei or particulate matter, which could otherwise act as unintended seeds and influence growth kinetics in subsequent runs. The phase and morphology of the CeO_2_ products from each cycle were characterized by XRD and SEM to evaluate the impact of recycling.

### 2.3. Characterization

The crystallographic phases of the samples were characterized by X-ray diffraction (XRD) on a DX-2700 diffractometer (Dandong Haoyuan Instrument Co., Ltd., Dandong, China) using Cu Kα radiation. The patterns were recorded in the 2θ range of 20–80° with a step size of 0.1°, operating at 30 kV and 25 mA. The morphology of the samples was examined by scanning electron microscopy (SEM) on a SEM5000 instrument (CIQTEK Co., Ltd., Hefei, China). Prior to observation, the samples were sputter-coated with a thin layer of platinum to enhance conductivity. Images were acquired in SE mode at an accelerating voltage of 5 kV.

## 3. Results and Discussion

### 3.1. Effect of Solvent Type and Binary Solvent System on Phase and Morphology

**Figure 1:**
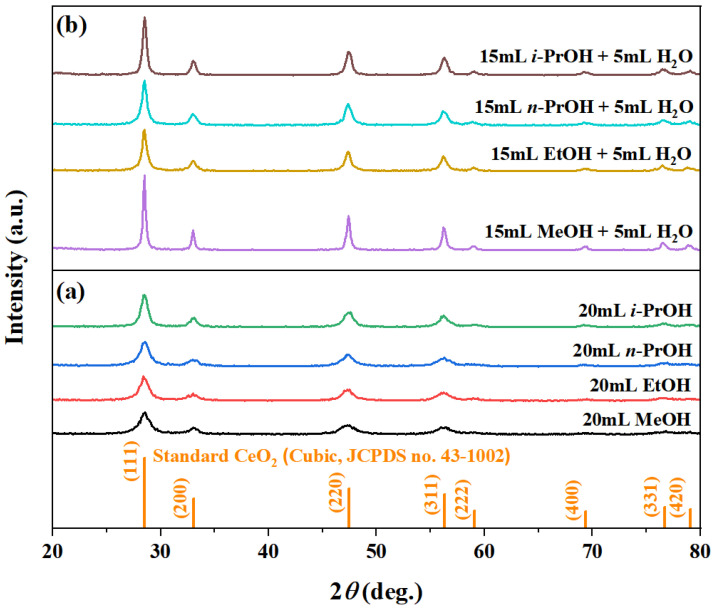
XRD patterns of the samples synthesized solvothermally at 180 °C for 12 h using (**a**) pure alcohols (20 mL) and (**b**) binary aqueous-mixed solutions (15 mL alcohol + 5 mL H_2_O). Alcohols: MeOH, EtOH, *n*-PrOH, and *i*-PrOH.

The XRD patterns in Figure 1 confirm the formation of pure-phase cubic CeO_2_ (JCPDS no. 43-1002) under all tested solvent conditions, whether using pure alcohols (20 mL of MeOH, EtOH, *n*-PrOH, or *i*-PrOH) or binary alcohol/H_2_O mixtures (15 mL alcohol + 5 mL H_2_O). The primary (111), (200), (220), and (311) diffraction peaks are prominent in every pattern (Figure 1a,b). Notably, the introduction of H_2_O significantly enhanced the diffraction intensity compared to the pure alcohol systems, indicating improved crystallinity (Table 2). Among the binary systems, MeOH/H_2_O yielded the highest peak intensity, followed by *i*-PrOH/H_2_O, whereas EtOH/H_2_O and *n*-PrOH/H_2_O produced lower intensities (Figure 1b). Although a lower alcohol boiling point (MeOH: 64.7 °C < EtOH: 78.3 °C < *i*-PrOH: 82.6 °C < *n*-PrOH: 97.1 °C) typically favors higher autogenous pressure and crystallization, the observed intensity trend does not follow this sequence. This suggests that solvent polarity and specific synergies with H_2_O collectively govern crystallization kinetics and final crystallinity.

In binary solvent systems, additional well-defined peaks corresponding to the (222), (400), (331), and (420) planes became visible (Figure 1b), implying that H_2_O not only promotes complete crystallization but may also modulate the relative growth rates of different crystal facets. The absence of impurity peaks underscores the efficacy of this one-step, solvent-controlled strategy. The crystallite sizes, calculated from the (111) peak broadening, increased from ~6.7–9.0 nm in pure alcohols to ~9.5–12.8 nm in the binary system. The phase purity and controlled crystallinity are crucial for functional performance, as demonstrated in analogous material systems where composition and morphology dictate properties [38].

**Figure 2:**
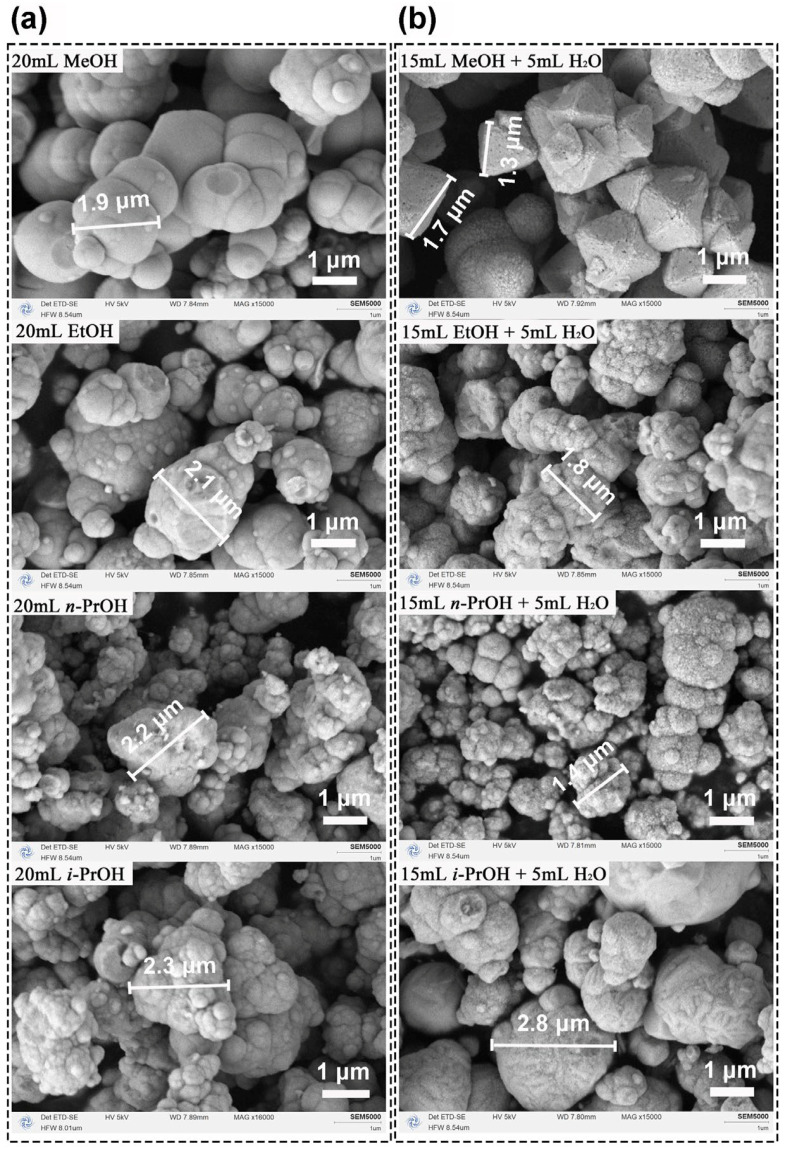
SEM images of the CeO_2_ samples synthesized solvothermally at 180 °C for 12 h using (**a**) pure alcohols (20 mL) and (**b**) binary aqueous-mixed solutions (15 mL alcohol + 5 mL H_2_O). Alcohols: MeOH, EtOH, *n*-PrOH, and *i*-PrOH.

Figure 2 presents the morphological evolution of CeO_2_ synthesized under different solvent systems. In pure alcohols (20 mL), all samples formed irregular aggregates with poor definition (Figure 2a). Specifically, pure MeOH yielded incomplete large spheroids mixed with fine particles, while EtOH, *n*-PrOH, and *i*-PrOH produced featureless aggregates, indicating ineffective morphological control (Table 2). Replacing 5 mL of alcohol with H_2_O (15 mL alcohol + 5 mL H_2_O) induced a clear morphological transition (Figure 2b). Although EtOH/H_2_O, *n*-PrOH/H_2_O, and *i*-PrOH/H_2_O still gave irregular aggregates, the MeOH/H_2_O system uniquely generated well-defined, uniform octahedra with sharp edges, consistent with the highest crystallinity and largest crystallite size (11.8 nm) observed in XRD (Table 2).

Combined with the XRD results (Figure 1), H_2_O not only enhances crystallinity but also critically directs morphological evolution. The exclusive formation of octahedra in the MeOH/H_2_O system highlights the solvent-specific promotion of oriented growth, resulting in a distinct faceted morphology. This aligns with established principles where crystal morphology governs surface properties, as seen in analogous material systems [39]. The contrast between well-defined octahedra in MeOH/H_2_O and irregular aggregates in other systems underscores the decisive role of solvent selection in morphology-controlled oxide synthesis.

### 3.2. Effect of MeOH/H_2_O Ratio on Phase and Morphology

**Figure 3:**
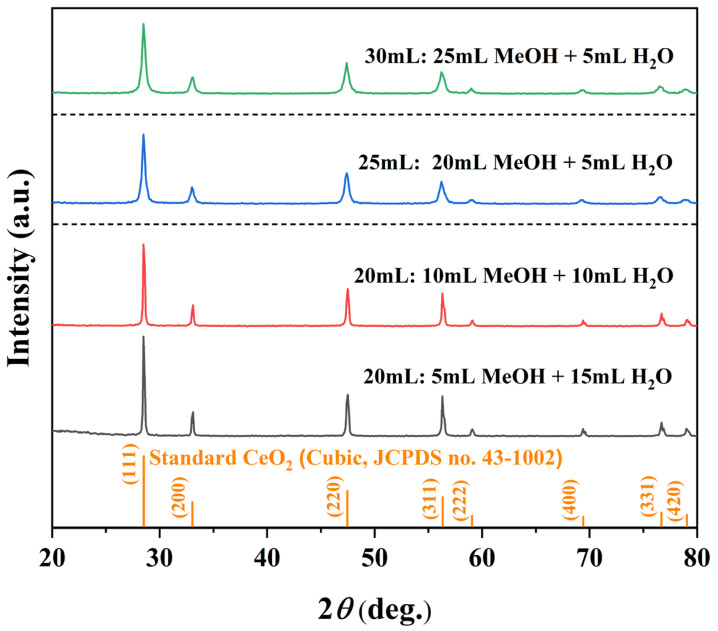
XRD patterns of samples synthesized solvothermally at 180 °C for 12 h under different MeOH/H_2_O solvent compositions: 5 mL MeOH + 15 mL H_2_O, 10 mL MeOH + 10 mL H_2_O, 20 mL MeOH + 5 mL H_2_O, and 25 mL MeOH + 5 mL H_2_O.

To elucidate how solvent composition governs CeO_2_ formation, we systematically varied the MeOH/H_2_O ratio following two experimental schemes. One maintained a fixed total solvent volume of 20 mL while varying the MeOH/H_2_O ratios at 5:15, 10:10, and 15:5 mL. Another kept the H_2_O volume constant at 5 mL and progressively increased the MeOH volume to 20 mL and 25 mL, corresponding to total volumes of 25 mL and 30 mL, respectively. All XRD patterns (Figure 3) confirm phase-pure cubic CeO_2_ (JCPDS no. 43-1002) without impurities. While variations in the MeOH/H_2_O ratio at a fixed 20 mL total volume caused only minor changes in peak intensity and width, increasing the total volume to 25 and 30 mL significantly reduced peak intensity and broadened the reflections (Table 3). This suggests that a higher solvent filling lowers the autogenous pressure and dilutes the precursor concentration, thereby impairing crystallinity and reducing crystallite size.

**Figure 4:**
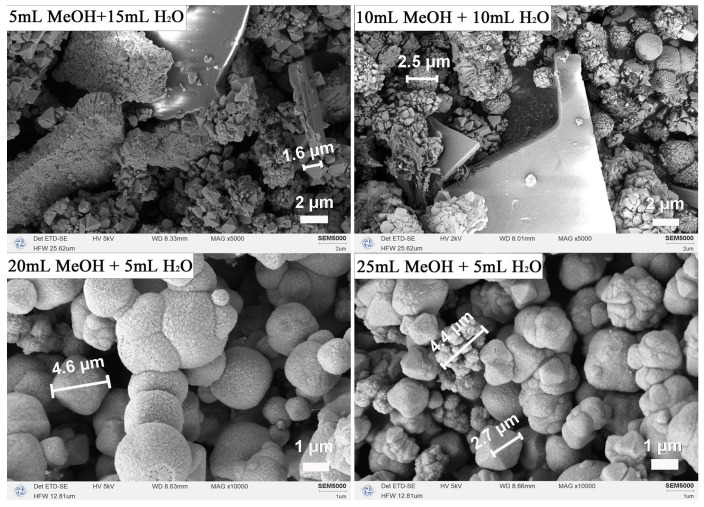
SEM images of CeO_2_ samples synthesized solvothermally at 180 °C for 12 h under different MeOH/H_2_O solvent compositions: 5 mL MeOH + 15 mL H_2_O, 10 mL MeOH + 10 mL H_2_O, 20 mL MeOH + 5 mL H_2_O, and 25 mL MeOH + 5 mL H_2_O.

Corresponding SEM images (Figure 4) reveal the morphological sensitivity to solvent composition. At a fixed 20 mL system, with higher H_2_O content (5:15 and 10:10) produced aggregated nano-octahedra, whereas the 15:5 ratio yielded well-separated, faceted micro-octahedra. When the total volume was increased to 25 mL and 30 mL (with constant 5 mL H_2_O), the products exhibited mixed octahedral/spherical shapes with progressively degraded edges. These observations demonstrate that phase purity is maintained across all conditions, the MeOH/H_2_O ratio and total volume critically control particle dispersion, edge definition, and overall morphological uniformity.

### 3.3. Effect of Solvothermal Duration on Crystallinity and Morphology

**Figure 5:**
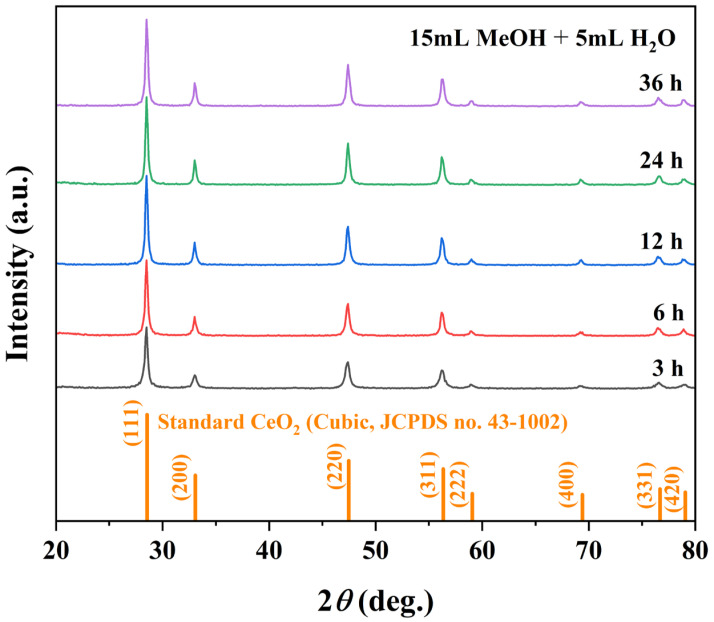
Crystallographic evolution of samples with solvothermal duration: XRD patterns of samples synthesized at 180 °C using a binary solvent of 15 mL MeOH + 5 mL H_2_O at different reaction times (3, 6, 12, 24, and 36 h).

The crystallization pathway of octahedral CeO_2_ was investigated by varying the solvothermal time (1–36 h) while maintaining the optimal solvent composition (15 mL MeOH + 5 mL H_2_O) and temperature (180 °C). XRD patterns in Figure 5 display that all samples except the 1 h product exhibit the characteristic peaks of phase-pure cubic CeO_2_ (JCPDS no. 43-1002). The 1 h sample yielded insufficient material for reliable XRD, indicating early-stage nucleation. From 3 to 12 h, the diffraction peak intensified and sharpened, reflecting progressive crystallinity improvement and grain growth, with the highest crystallinity achieved at 12 h (Table 4). Peak profiles stabilized at 24 h, suggesting completion of primary crystallization. After 36 h, peaks broadening and a slight decrease in the (111) intensity indicate reduced crystallite size and the onset of surface dissolution or Ostwald ripening.

**Figure 6:**
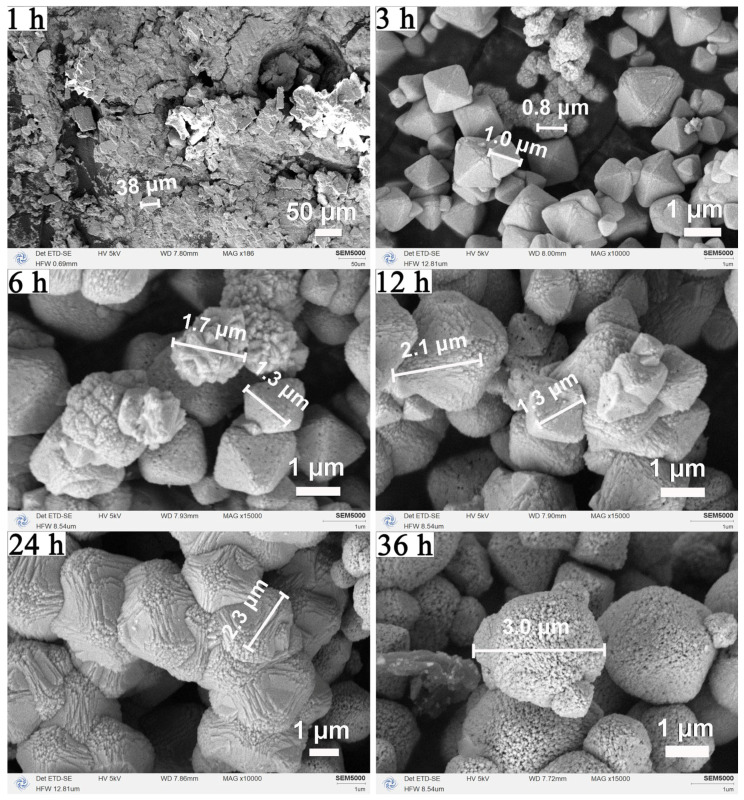
Morphological evolution of CeO_2_ with solvothermal duration: SEM images of CeO_2_ samples synthesized at 180 °C using a binary solvent of 15 mL MeOH and 5 mL H_2_O at different reaction times (1, 3, 6, 12, 24, and 36 h).

The corresponding morphological evolution, as revealed by SEM in Figure 6, further supports this crystallization pathway. At 1 h, only undefined plate-like structures were observed. By 3 h, a mixture of nano- and micro-sized octahedra appeared. At 12 h, well-faceted micro-octahedra predominated, consistent with the optimal crystallinity seen in XRD. Crystal size continued to increase until 24 h. After 36 h, surface pitting and cavities became evident, confirming partial dissolution and aligning with the XRD peak broadening. Collectively, the results delineate a clear time-dependent trajectory: nucleation and crystallization (1–12 h), crystal growth and maturation (12–24 h), and surface restructuring or partial dissolution under extended conditions (>24 h). This understanding provides crucial guidance for selecting the optimal reaction window to obtain CeO_2_ octahedra with high crystallinity and well-defined morphology.

### 3.4. Recycling and Reuse of Solvothermal Mother Liquor

**Figure 7:**
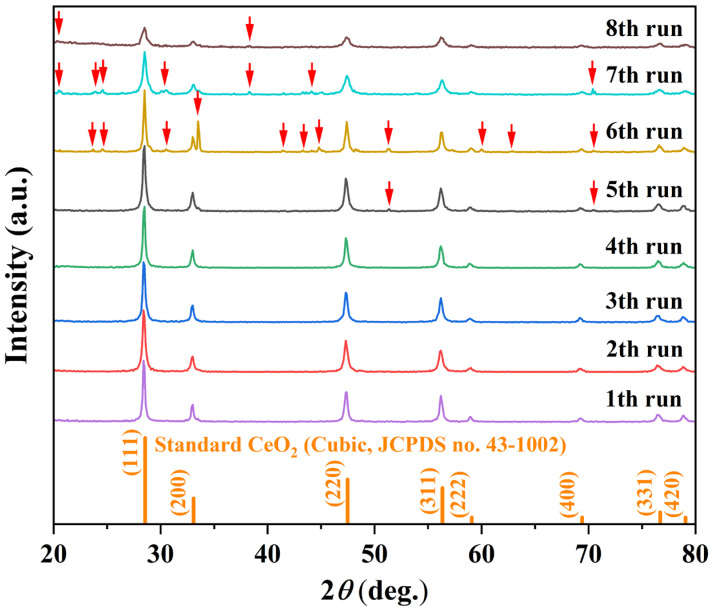
Crystallographic evolution of the samples synthesized via recycled solvothermal mother liquor: XRD patterns of samples obtained after eight successive recycling cycles (180 °C, 12 h per cycle).

To align with green chemistry principles and enhance the sustainability of the synthesis [40], the solvothermal mother liquor was recycled over multiple cycles under fixed conditions (180 °C, 12 h, initial solvent: 15 mL MeOH + 5 mL H_2_O). Figure 7 displays the XRD patterns of CeO_2_ synthesized over successive recycling rounds. During the first four cycles, all diffraction peaks correspond exclusively to the cubic fluorite structure of CeO_2_, with no detectable secondary phases. The consistency in peak position, intensity, and profile indicates excellent retention of crystallinity and phase purity throughout these initial reuses, which is corroborated by the stable crystallite size (12.4–15.7 nm) and high relative crystallinity (65.3–65.8%) recorded in Table 4. However, upon the fifth cycle, two weak impurity peaks emerged at 2θ = 51.3° and 70.4°. By the eighth cycle, additional impurity peaks became evident (marked by red arrows in Figure 7), accompanied by a discernible decrease in the intensity of the characteristic CeO_2_ reflections. These emerging peaks are attributed to orthorhombic CeCO_3_OH (JCPDS no. 41-0013) and hexagonal CeCO_3_OH (JCPDS no. 52-0352). The appearance of these impurities suggests the gradual accumulation of dissolved species or reaction byproducts in the recycled mother liquor, which ultimately interfere with the crystallization process and promote the formation of secondary phases, consistent with the observed loss of well-defined octahedral morphology and the onset of irregular aggregates noted in Table 4 for cycles 5–8.

**Figure 8:**
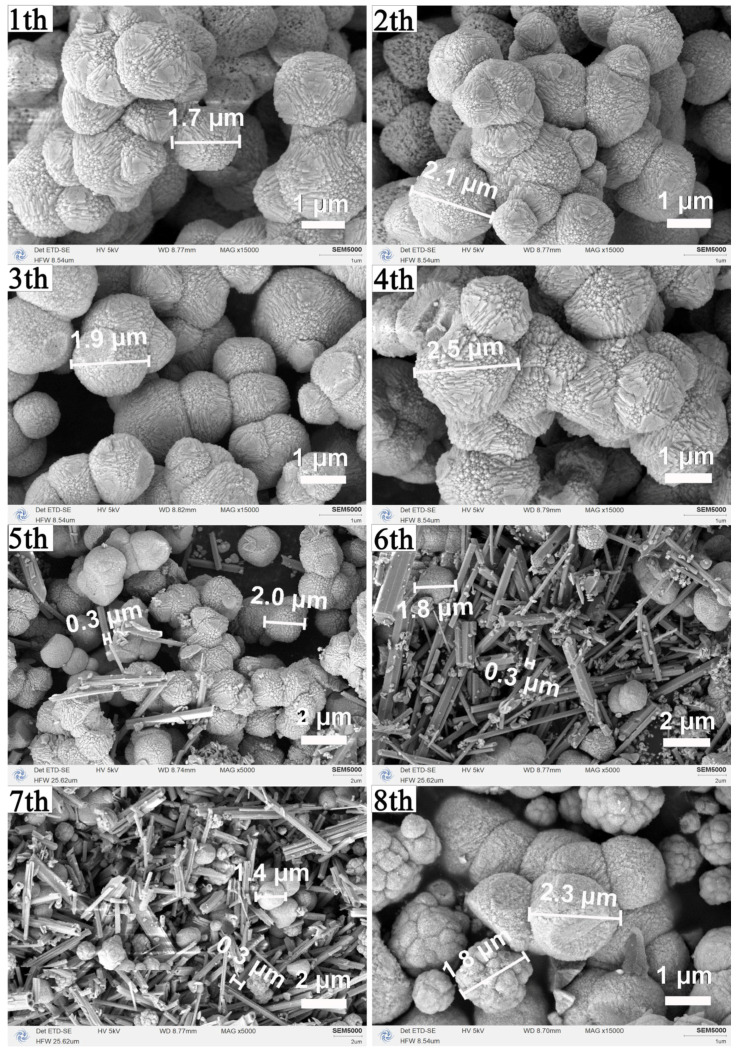
Morphological evolution of the samples synthesized via recycled solvothermal mother liquor: SEM images of samples obtained after eight successive recycling cycles (180 °C, 12 h per cycle).

SEM images (Figure 8) reveal that uniform micron-scale octahedra are maintained through four cycles. From the fifth cycle onward, prismatic and nanoparticulate impurities appear, and by the eighth cycle, the product consists mainly of angular aggregates and near-spherical particles, consistent with the XRD detection of CeCO_3_OH. Together, XRD and SEM confirm that the mother liquor can be effectively recycled four times without degrading the phase purity or the octahedral morphology of CeO_2_. Beyond this point, accumulated byproducts lead to impurity formation and loss of morphological control. This closed-loop recycling strategy highlights the practical sustainability of the proposed synthesis route.

### 3.5. Mechanism Discussion

#### 3.5.1. Formation Mechanism of CeO_2_

Based on the experimental results, the formation and evolution of CeO_2_ in the solvothermal reaction system can be described through the following mechanisms. In both pure alcohol and alcohol/H_2_O binary systems, CeO_2_ synthesis proceeds through a coordinated sequence of oxidation, catalysis, hydrolysis, and dissolution-recrystallization. Initially, Ce^3+^ ions from Ce(NO_3_)_3_·6H_2_O interact with monohydric alcohols (C_n_H_2n+1_OH) in the presence of dissolved molecular oxygen, initiating the oxidation and nucleation of CeO_2_. Continued oxygen participation facilitates the formation of intermediate cerium species, ultimately leading to CeO_2_ crystallization (Equations (1)–(3)). The oxygen originates from both trapped air in the reactor and oxygen dissolved in the solvent.

(1) $${\mathrm{Ce}}^{3+}{+3(\mathrm{HOC}}_{n}H_{2n+1})\;⇄{\mathrm{Ce}(\mathrm{OC}}_{n}H_{2n+1}{)}_{3}+3H^{+}$$

(2) $${\mathrm{Ce}(\mathrm{OC}}_{n}H_{2n+1}{)}_{3}{+3(\mathrm{HOC}}_{n}H_{2n+1})\;⇄{\;\mathrm{Ce}(\mathrm{OH})}_{3}{+3(C}_{n}H_{2n+1}{\mathrm{OC}}_{n}H_{2n+1})$$

(3) $${\;4\mathrm{Ce}(\mathrm{OH})}_{3}{+O}_{2}⇄{\;4\mathrm{CeO}}_{2}+6H_{2}O$$

Once formed, CeO_2_ nanocrystals serve as catalysts for the subsequent oxidation of the alcohol solvent. The C_n_H_2n+1_OH can be oxidized to carboxylic acids (H_2n−1_C_n_OOH) (Equation (4)). H_2_O plays a crucial mediating role by promoting the hydrolysis and solvation of Ce^3+^ ions, thereby influencing the reaction environment (Equation (5)). Concurrently, nitrate ions (NO_3_^−^) from the cerium precursor (Ce(NO_3_)_3_·6H_2_O) serve as an intrinsic oxidant under solvothermal conditions, contributing to the oxidation of Ce^3+^ and the release of gaseous products (Equation (6)) [41].

(4) $${\mathrm{HOC}}_{n}H_{2n+1}+O_{2}\;\overset{{\mathrm{CeO}}_{2}}{⇄}{\;H}_{2n-1}C_{n}\mathrm{OOH}+H_{2}O$$

(5) $$H_{2n-1}C_{n}\mathrm{OOH}\;\overset{\mathrm{Ionization}}{\underset{H_{2}O}{⇄}}H_{2n-1}C_{n}{\mathrm{OO}}^{-}+H^{+}$$

(6) $${\;2\mathrm{Ce}(\mathrm{OH})}_{3}+2{\mathrm{NO}}_{3}^{-}+2H^{+}⇄{\;2\mathrm{CeO}}_{2}{+2\mathrm{NO}}_{2}+4H_{2}O$$

The organic acids (H_2n+1_C_n_OOH) generated in situ, such as formic acid or acetic acid, participate in a reversible dissolution reaction with CeO_2_ (Equation (7)). This acid-mediated dissolution-recrystallization process is central to the morphological evolution of CeO_2_, promoting Ostwald ripening and enabling the transformation from irregular aggregates into well-defined octahedra in the MeOH/H_2_O system. This mechanism aligns with the observed time-dependent enhancement in crystallinity and particle uniformity between 3 and 24 h, as well as the surface restructuring observed beyond 24 h in Figure 6.

(7) $${4\mathrm{CeO}}_{2}+12H^{+}⇄{4\mathrm{Ce}}^{3+}+6H_{2}O+O_{2}$$

The dual role of MeOH in this system warrants further discussion, particularly in the context of morphological control. It is well-recognized that alcohol molecules, with their hydroxyl groups (−OH), can adsorb onto oxide surfaces and influence crystal growth kinetics. In our case, MeOH (and its in situ oxidation products like formaldehyde or formic acid) can indeed act as in situ-generated surface modifiers. They may preferentially adsorb onto specific crystallographic facets of CeO_2_, thereby modulating relative growth rates and contributing to the final octahedral morphology.

This mechanism, however, is fundamentally distinct from conventional templating or surfactant-assisted syntheses. In those methods, externally added agents (e.g., CTAB, polymers, or anionic capping agents) are introduced a priori with the primary intent of directing morphology, and their post-synthesis removal is often mandatory. In contrast, our strategy employs only the solvent itself as the reaction medium and the source of these modulating species. No auxiliary templates, surfactants, or anions are added. The surface-modifying species are generated in situ from the solvent during the reaction, are integral to the solvent matrix, and do not require a separate removal step. Therefore, while acknowledging the active role of MeOH-derived species in morphology control, we emphasize that our approach achieves shape control without the introduction of any foreign template or surfactant additives, relying solely on the intrinsic properties and reactivity of the binary solvent system.

#### 3.5.2. Mechanism of Impurity Formation During Solvent Recycling

During the repeated recycling of the solvothermal mother liquor, the accumulation of carbonaceous species leads to the gradual formation of crystalline impurities, primarily cerium carbonate hydroxide (CeCO_3_OH), including orthorhombic and hexagonal CeCO_3_OH. With increasing cycles (≥5), formate or other organic anions accumulated in the mother liquor undergo decomposition or conversion to carbonate ions (Equation (8)). These carbonate ions then react with residual Ce^3+^ and hydroxide ions in the recycled solvent to precipitate as CeCO_3_OH (Equations (9) and (10)) [42]. Although CeCO_3_OH may partially decompose under prolonged heating, under the given recycling conditions, it persists as an impurity phase. The presence of these impurities coincides with the deterioration of octahedral morphology and the emergence of irregular aggregates, confirming that the solvent can be effectively recycled only up to four cycles without compromising product purity or morphology.

(8) $${\mathrm{CH}}_{3}{\mathrm{OH}+6\mathrm{CeO}}_{2}+16H^{+}⇄\;6{\mathrm{Ce}}^{3+}+{\mathrm{CO}}_{3}^{2-}+10H_{2}O$$

(9) $${{[\mathrm{Ce}(H}_{2}{O)}_{n}]}^{3+}{+H}_{2}O\;⇄{{[\mathrm{Ce}(\mathrm{OH})(H}_{2}{O)}_{n-1}]}^{2+}{+H}_{3}O^{+}$$

(10) $${{[\mathrm{Ce}(\mathrm{OH})(H}_{2}{O)}_{n-1}]}^{2+}+{\mathrm{CO}}_{3}^{2-}⇄{\mathrm{CeCO}}_{3}{\mathrm{OH}+(n-1)H}_{2}O$$

## 4. Conclusions

A facile one-step solvothermal strategy has been developed to synthesize octahedral CeO_2_ without the addition of external templates, surfactants, or post-calcination. Employing only Ce(NO_3_)_3_·6H_2_O in a binary MeOH/H_2_O solvent system under optimized conditions (15 mL MeOH + 5 mL H_2_O, 180 °C, 3–24 h) yields pure-phase cubic CeO_2_ with well-defined octahedral morphology. Solvent parameters critically govern morphological evolution: while variations in the MeOH/H_2_O ratio (5–25 mL) and total solvent volume (20–30 mL) preserve phase purity and crystallinity, they strongly influence particle aggregation and edge definition. Prolonged reaction beyond 24 h induces partial surface dissolution while maintaining phase integrity. The solvothermal mother liquor can be recycled four times without degrading phase purity or octahedral morphology, demonstrating a closed-loop sustainable process. This solvent-only approach, which requires no auxiliary additives, eliminates hazardous byproducts and complex purification, offering a green and practical route for the morphology-controlled CeO_2_ synthesis with minimal waste.

## Figures and Tables

**Table 1:** Summary of solvent compositions and reaction conditions for the synthesis of CeO_2_.

Experiment Type	Solvent Composition	Total Volume (mL)	Ce(NO_3_)_3_·6H_2_O	Conditions
Pure Organic Solvent Systems	20 mL MeOH	20	4 mmol	180 °C, 12 h
	20 mL EtOH	20	4 mmol	180 °C, 12 h
	20 mL *n*-PrOH	20	4 mmol	180 °C, 12 h
	20 mL *i*-PrOH	20	4 mmol	180 °C, 12 h
Binary Solvent Systems	15 mL MeOH + 5 mL H_2_O	20	4 mmol	180 °C, 12 h
	15 mL EtOH + 5 mL H_2_O	20	4 mmol	180 °C, 12 h
	15 mL *n*-PrOH + 5 mL H_2_O	20	4 mmol	180 °C, 12 h
	15 mL *i*-PrOH + 5 mL H_2_O	20	4 mmol	180 °C, 12 h
MeOH/H_2_O Ratio Study	5 mL MeOH + 15 mL H_2_O	20	4 mmol	180 °C, 12 h
	10 mL MeOH + 10 mL H_2_O	20	4 mmol	180 °C, 12 h
	15 mL MeOH + 5 mL H_2_O	20	4 mmol	180 °C, 12 h
MeOH/H_2_O Volume Study	20 mL MeOH + 5 mL H_2_O	25	4 mmol	180 °C, 12 h
	25 mL MeOH + 5 mL H_2_O	30	4 mmol	180 °C, 12 h
Recycling Study	Recovered mother liquor (Initial: 15 mL MeOH + 5 mL H_2_O)	20	4 mmol per cycle	180 °C, 12 h per cycle

**Table 2:** Structural and morphological parameters of CeO_2_ synthesized in different solvent systems.

Solvent System	Crystallite Size (nm)	Relative Crystallinity (%)	Particle Size Range (μm)	Dominant Morphology (from SEM)
**Pure Alcohols (20 mL)**				
MeOH	6.7	41.2	~1.9 (Diameter)	Irregular spherical aggregates
EtOH	6.9	49.2	~2.1 (Diameter)	Irregular spherical aggregates
*n*-PrOH	7.2	48.3	~2.2 (Diameter)	Irregular spherical aggregates
*i*-PrOH	9.0	60.3	~2.3 (Diameter)	Irregular spherical aggregates
**Binary Solvents (15 mL alcohol/5 mL H_2_O)**				
MeOH/H_2_O	11.8	62.7	~1.3/1.7 (Edge length)	Well-defined octahedra
EtOH/H_2_O	10.8	57.0	~1.8 (Diameter)	Irregular spherical aggregates
*n*-PrOH/H_2_O	9.5	59.2	~1.4 (Diameter)	Irregular spherical aggregates
*i*-PrOH/H_2_O	12.8	61.1	~2.8 (Diameter)	Irregular spherical aggregates

**Note:** Crystallite Size (nm) was calculated from the full width at half maximum (FWHM) of the (111) diffraction peak using the Scherrer equation.

**Table 3:** Effect of MeOH/H_2_O volume ratio on the structural parameters of CeO_2_.

MeOH/H_2_O (mL/mL)	Total Volume (mL)	Crystallite Size (nm)	Relative Crystallinity (%)	Particle Size Range (μm)	Dominant Morphology (from SEM)
5/15	20	13.2	61.9	~1.6 (Edge length)	Nano-octahedra aggregates
10/10	20	16.4	63.5	~2.5 (Diameter)	Nano-octahedra aggregates
20/5	25	13.4	63.0	~14.6 (Diameter)	Octahedra with degraded edges
25/5	30	11.3	55.9	~2.7 (Edge length)/~4.4 (Diameter)	Octahedra with degraded edges

**Table 4:** Evolution of structural parameters and morphology of CeO_2_ under optimal solvent composition (15 mL MeOH/5 mL H_2_O) with varying reaction time and upon mother liquor recycling.

Condition	Variable	Crystallite Size (nm)	Relative Crystallinity (%)	Particle Size Range (μm)	Dominant Morphology (from SEM)
Reaction Time	1 h	/	/	~38 (Diameter)	Initial nanoplate aggregates
	3 h	13.5	62.3	~1.0 (Edge length)/~0.8 (Diameter)	Nano- and micro-sized octahedra
	6 h	12.4	62.6	~1.3 (Edge length)/~1.7 (Diameter)	Growth of micro-octahedra
	12 h	11.8	62.7	~1.3/2.1 (Edge length)	Well-faceted micro-octahedra
	24 h	16.6	65.4	~2.3 (Edge length)	Larger octahedra
	36 h	12.9	63.7	~3.0 (Diameter)	Surface pitting, onset of dissolution
Recycling Cycle	Cycles 1–4	12.4/13.7/12.8/15.7	65.3/64.2/65.6/65.8	~1.7/2.1 1.9/2.5 (Diameter)	Uniform micron-sized octahedra
	Cycles 5–7	/	/	/	Appearance of prismatic/nanoparticles
	Cycles 8	/	/	/	Angular to spherical aggregates

## Data Availability

Data are contained within the article.
